# Early brain injury after aneurysmal subarachnoid hemorrhage: a multimodal neuromonitoring study

**DOI:** 10.1186/s13054-015-0809-9

**Published:** 2015-03-09

**Authors:** Raimund Helbok, Alois Josef Schiefecker, Ronny Beer, Anelia Dietmann, Ana Patrícia Antunes, Florian Sohm, Marlene Fischer, Werner Oskar Hackl, Paul Rhomberg, Peter Lackner, Bettina Pfausler, Claudius Thomé, Christian Humpel, Erich Schmutzhard

**Affiliations:** Neurological Intensive Care Unit, Department of Neurology, Medical University of Innsbruck, Anichstreet 35, 6020 Innsbruck, Austria; Department of Neurosciences, Santa Maria Hospital, Hospital de Santa Maria, 1649-028 Lisbon, Portugal; Department of Neurosurgery, Innsbruck Medical University, Anichstreet 35, 6020 Innsbruck, Austria; Institute of Biomedical Informatics, UMIT-University for Health Sciences, Medical Informatics and Technology, Eduard Wallnöfer-Zentrum I, 6060 Hall in Tirol, Austria; Department of Radiology, Innsbruck Medical University, Anichstreet 35, 6020 Innsbruck, Austria; Department of Psychiatry and Psychotherapy, Medical University Innsbruck, Anichstreet 35, 6020 Innsbruck, Austria

## Abstract

**Introduction:**

There is a substantial amount of evidence from animal models that early brain injury (EBI) may play an important role for secondary brain injury after aneurysmal subarachnoid hemorrhage (aSAH). Cerebral microdialysis (CMD) allows online measurement of brain metabolites, including the pro-inflammatory cytokine interleukin-6 (IL-6) and matrix metalloproteinase-9 (MMP-9), which is indicative for disruption of the blood-brain barrier.

**Methods:**

Twenty-six consecutive poor-grade aSAH patients with multimodal neuromonitoring were analyzed for brain hemodynamic and metabolic changes, including CMD-IL-6 and CMD-MMP-9 levels. Statistical analysis was performed by using a generalized estimating equation with an autoregressive function.

**Results:**

The baseline cerebral metabolic profile revealed brain metabolic distress and an excitatory response which improved over the following 5 days (*P* <0.001). Brain tissue hypoxia (brain tissue oxygen tension of less than 20 mm Hg) was common (more than 60% of patients) in the first 24 hours of neuromonitoring and improved thereafter (*P* <0.05). Baseline CMD-IL-6 and CMD-MMP-9 levels were elevated in all patients (median = 4,059 pg/mL, interquartile range (IQR) = 1,316 to 12,456 pg/mL and median = 851 pg/mL, IQR = 98 to 25,860 pg/mL) and significantly decreased over days (*P* <0.05). A higher pro-inflammatory response was associated with the development of delayed cerebral ischemia (*P* = 0.04), whereas admission disease severity and early brain tissue hypoxia were associated with higher CMD-MMP-9 levels (*P* <0.03). Brain metabolic distress and increased IL-6 levels were associated with poor functional outcome (modified Rankin Scale of more than 3, *P* ≤0.01). All models were adjusted for probe location, aneurysm securing procedure, and disease severity as appropriate.

**Conclusions:**

Multimodal neuromonitoring techniques allow insight into pathophysiologic changes in the early phase after aSAH. The results may be used as endpoints for future interventions targeting EBI in poor-grade aSAH patients.

## Introduction

Aneurysmal subarachnoid hemorrhage (aSAH) is a medical emergency with high mortality and morbidity [1,2]. The contribution of delayed cerebral ischemia (DCI) on outcome is undisputed, although the relief of cerebral vasospasm in the subacute phase after aSAH failed to improve functional outcome [3]. Despite advances in neurointensive care, the underlying mechanisms of secondary brain injury remain incompletely understood. Animal data support the importance of pathophysiologic mechanisms in the very early phase after SAH with changes including early vasospasm, inflammation, and global cerebral edema (GCE) [4]. Early brain injury (EBI) is now being recognized as an important cause of mortality and disability after SAH in humans and may be associated with DCI [5]. So far, pathophysiologic mechanisms related to EBI are under-investigated in humans, and no treatment is available to adequately address these processes. Although difficulties exist in translating findings from the experimental setting to the patients’ bedside, animal data convincingly provide evidence of neuronal damage within minutes after SAH triggered by brain tissue hypoxia, cerebral inflammation, blood-brain barrier (BBB) breakdown, and others [4]. Monitoring of such events in the very early phase in humans is challenging; however, invasive multimodal neuromonitoring devices allow continuous data acquisition for intracranial pressure (ICP), brain tissue oxygen tension (P_bt_O_2_), cerebral blood flow, and at least hourly information on brain metabolism already within the first 24 hours after aneurysm bleeding [6]. Using multimodal neuromonitoring data, we previously showed derangement in cerebral metabolism and increased episodes of brain tissue hypoxia in the first days after aSAH in patients with radiologic evidence of GCE compared with those without GCE [7]. The pro-inflammatory cytokine interleukin-6 (IL-6) in the cerebral microdialysate as a marker for neuroinflammation has been shown to be associated with DCI and unfavorable outcome following aSAH [8-10]. Matrix metalloproteinases (MMPs) are involved in vascular remodeling, neuroinflammation, BBB breakdown, and neuronal apoptosis [11-13]. In the experimental setting, MMP-9 potentiates EBI and was associated with apoptosis of hippocampal neurons of rats [11]. In patients with SAH, MMP-9 was associated with disease severity and the development of cerebral vasospasm [14,15].

The goal of the current study was to study pathophysiological events involved in the development of EBI in poor-grade aSAH patients by investigating brain hemodynamics—ICP, cerebral perfusion pressure (CPP), and P_bt_O_2_—and brain metabolic changes in combination with the local inflammatory response by cerebral microdialysis (CMD)-IL-6 and the function of the BBB by CMD-MMP-9 in the brain extracellular fluid. We intended to focus on the early phase after aSAH and relate these findings to clinical course and outcome.

## Methods

### Patient selection and care

Between 2010 and 2012, 26 consecutive poor-grade aSAH patients admitted to the Neurological Intensive Care Unit at Innsbruck Medical University requiring multimodal neuromonitoring (Glasgow Coma Scale Score of not more than 8) were studied. One third of our patients presented with Hunt and Hess (H&H) grade 1 to 3 at hospital admission and were eligible for neuromonitoring secondary to early neurological worsening (n = 4/26, 15%) or secondary brain swelling (n = 4/26, 15%). The clinical care of aSAH patients conforms to guidelines set forth by the American Heart Association [16]. All patients were followed with transcranial doppler sonography (TCD) (DWL Doppler-Box system; Compumedics, Singen, Germany) and received continuous intravenous nimodipine. All patients were comatose and treated with continuous sufentanil or ketamine and midazolam drips (or both) to facilitate mechanical ventilation. Acceleration of TCD mean blood flow velocity (mBFV) of more than 120 cm/s in the middle or anterior cerebral artery or daily change in mean TCD velocities greater than 50 cm/s was suggestive of cerebral vasospasm. A catheter cerebral angiogram was performed in patients with severe vasospasm (TCD-mBFV of more than 200 cm/s) refractory to hypertensive therapy (CPP target of more than 80 mm Hg) and treated with intra-arterial nimodipine. Cerebral infarction from DCI was defined as appearance of new infarction on head computed tomography (CT) that was judged by an independent radiologist (PR) to be not attributed to other causes [17]. GCE was defined by an independent neuroradiologist (PR) on the basis of the initial head CT scan as previously described: (1) complete or near-complete effacement of the hemispheric sulci and basal cisterns and (2) bilateral and extensive disruption of the hemispheric gray-white matter junction at the level of the centrum semiovale, which was due to either blurring or diffuse peripheral ‘finger-like’ extension of the transition zone between gray and white matter [18].

### Data collection, neuromonitoring, and ethical approval

All admission variables and hospital complications were prospectively recorded in our institutional SAH outcome database, as approved by the local ethics committee (Medical University Innsbruck, AN3898 285/4.8, AM4091-292/4.6). Functional outcome was assessed at 3 months post-bleeding by using the modified Rankin Scale (mRS), and poor outcome was defined as mRS of more than 3. Based on clinical and imaging criteria, patients underwent monitoring of cerebral metabolism, P_bt_O_2_, and ICP according to the local institutional protocol, which is in compliance with the Helsinki Declaration and has been approved by the local ethics committee (UN3898 285/4.8). Written informed consent was obtained according to federal regulations. Through a right frontal burr hole, a triple-lumen bolt was affixed to insert a Licox Clark-type probe (Integra Licox Brain Oxygen Monitoring; Integra NeuroSciences, Ratingen, Germany) and an ICP parenchymal probe (Neurovent_P-Temp; Raumedic, Münchberg, Germany). In addition, a high-cutoff brain microdialysis catheter (CMA-71; M-Dialysis, Stockholm, Sweden) was tunneled and inserted into the brain parenchyma for hourly assessment of brain metabolism. Isotonic perfusion fluid (Perfusion Fluid CNS; M-Dialysis, Stockholm, Sweden) was pumped through the system at a flow rate of 0.3 μL/minute. Hourly samples were analyzed with CMA 600 and Iscus^flex^ (M-Dialysis, Stockholm, Sweden) for cerebral extracellular glucose, pyruvate, lactate, and glutamate concentrations. At least 1 hour passed after the insertion of the probe and the start of the sampling in order to allow for normalization of changes due to probe insertion. After routine analysis, samples were kept at −80°C. Monitoring devices were inserted into the parenchyma of the vascular territory of the parent vessel of the aneurysm and the location confirmed by brain CT immediately after the procedure and classified as placed in morphologically ‘normal’ tissue or ‘perilesional’ (less than 1 cm from the lesion). Brain metabolic distress was defined as lactate-to-pyruvate ratio (LPR) of more than 40, and brain tissue hypoxia as P_bt_O_2_ of less than 20 mm Hg [19]. All continuously measured parameters were saved on a 3-minute average interval by using our patient data management system (Centricity* Critical Care 7.0 SP2; GE Healthcare Information Technologies, Dornstadt, Germany).

### Analytical methods

In all patients, IL-6 and MMP-9 levels could be measured in a single microdialysis sample collected over a period of one hour. Analysis of CMD-IL-6 and CMD-MMP-9 was performed by enzyme-linked immunosorbent assays as described by the manufacturer (Aushon Custom Chemiluminescent Array Kit: 2-plex; Aushon BioSystems, Billerica, MA, USA). Calibrated protein standards (50 μL) and cerebral microdialysate (6 μL) diluted in 50 μL of buffer were added to pre-coated wells and incubated for 150 minutes. The wells were incubated for 30 minutes with biotinylated antibodies and then 30 minutes with streptavidin-horseradish peroxidase conjugate. Finally, the SuperSignal Chemiluminescent Substrate was added. All incubation steps were performed on a shaker at room temperature, and all wells were washed after every incubation step. The luminescent signal was detected by using a CCD (charge-coupled device) imaging and analysis system. The concentration of each sample was quantified by comparing the spot intensities to the corresponding standard curves calculated from the standard sample results by using SearchLight® Analyst Software (Aushon BioSystems). CMD-IL-6 detection limit was 0.4 pg/mL.

### Statistical analysis

**Figure 1:**
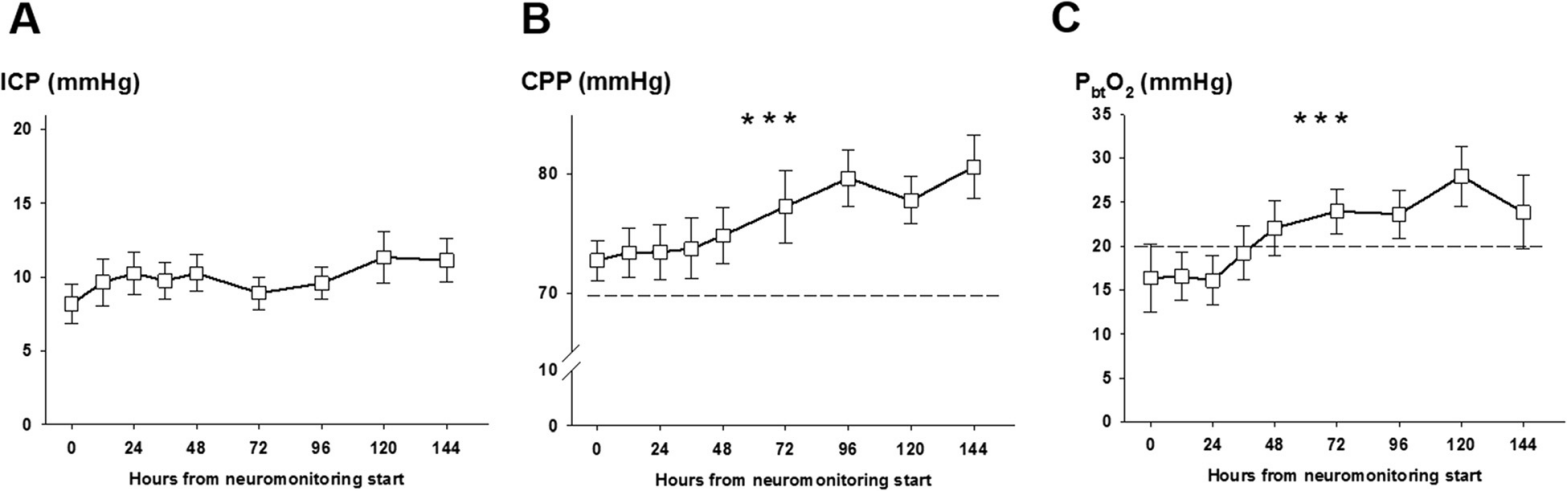
**Mean intracranial pressure (ICP), cerebral perfusion pressure (CPP), and brain tissue oxygen tension (P**_**bt**_ **O**_**2**_ **) of 26 aneurysmal subarachnoid hemorrhage patients at given time points after neuromonitoring was started. (A)** Mean (standard error of the mean) ICP over the first 144 hours of neuromonitoring. Significant increase of CPP **(B)** and P_btO2_ **(C)** over time. ***P <0.001. Dashed lines indicate commonly used cutoffs (CPP at 70 mm Hg and P_btO2_ at 20 mm Hg). Values are presented as mean ± standard error of the mean.

**Figure 2:**
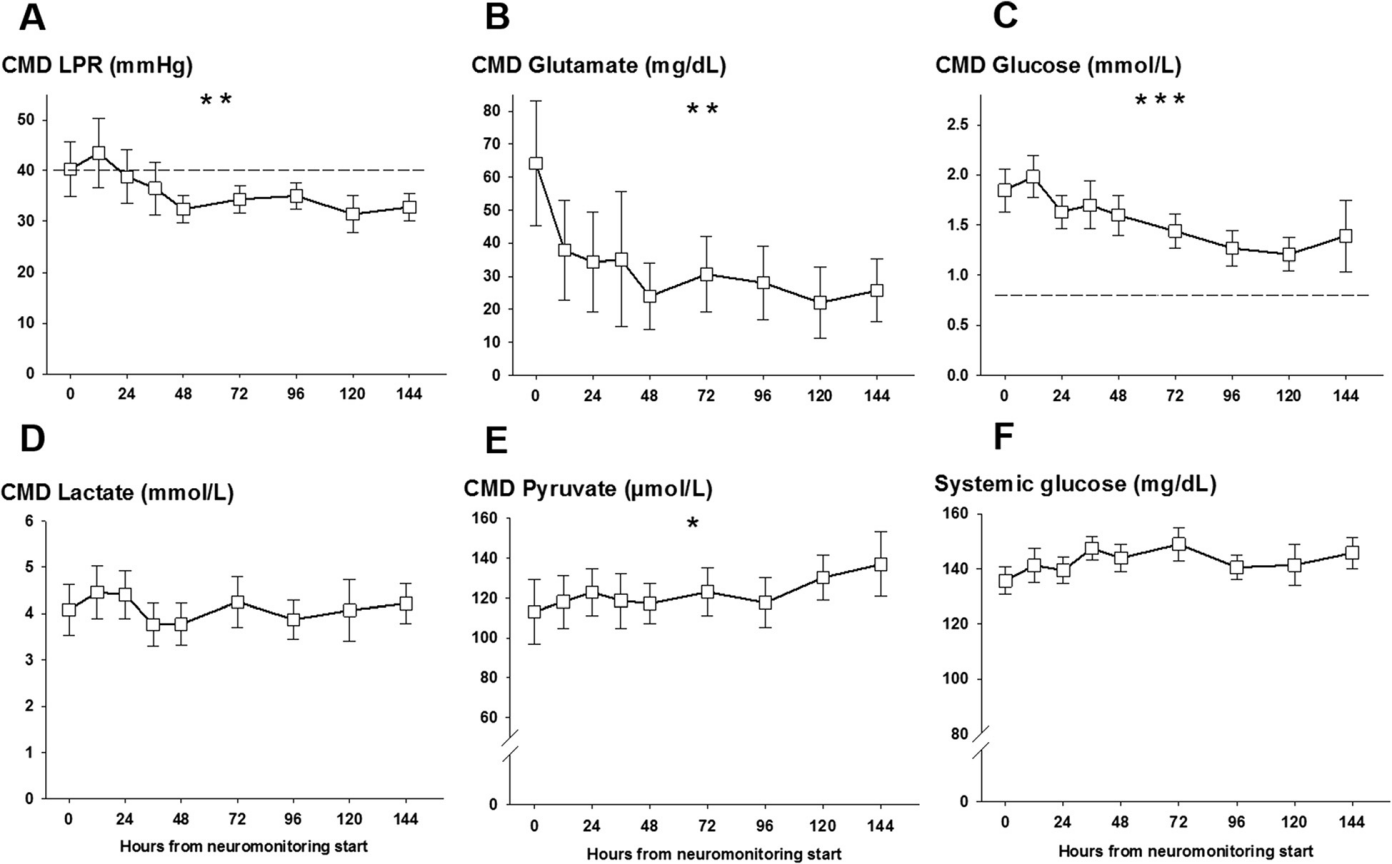
**Mean lactate-to-pyruvate ratio (LPR), glutamate, glucose, lactate, and pyruvate levels in the cerebral microdialysate and systemic glucose levels of 26 aneurysmal subarachnoid hemorrhage patients at given time points after neuromonitoring was started.** Significant decrease of CMD-LPR **(A)**, CMD-glutamate **(B)**, and CMD-glucose **(C)**. **P <0.01, ***P <0.001. Dashed lines indicate commonly used cutoffs (CMD-LPR at 40 and CMD-glucose at 0.7 mmol/L). Panel **(D-F)** Shows CMD-lactate, CMD-pyruvate and systemic glucose levels over time with significant increase of CMD-pyruvate levels **(E)** (*P <0.05). CMD-lactate **(D)** and systemic glucose levels **(F)** remained stable over the neuromonitoring time. Values are presented as mean ± standard error of the mean.

**Figure 3:**
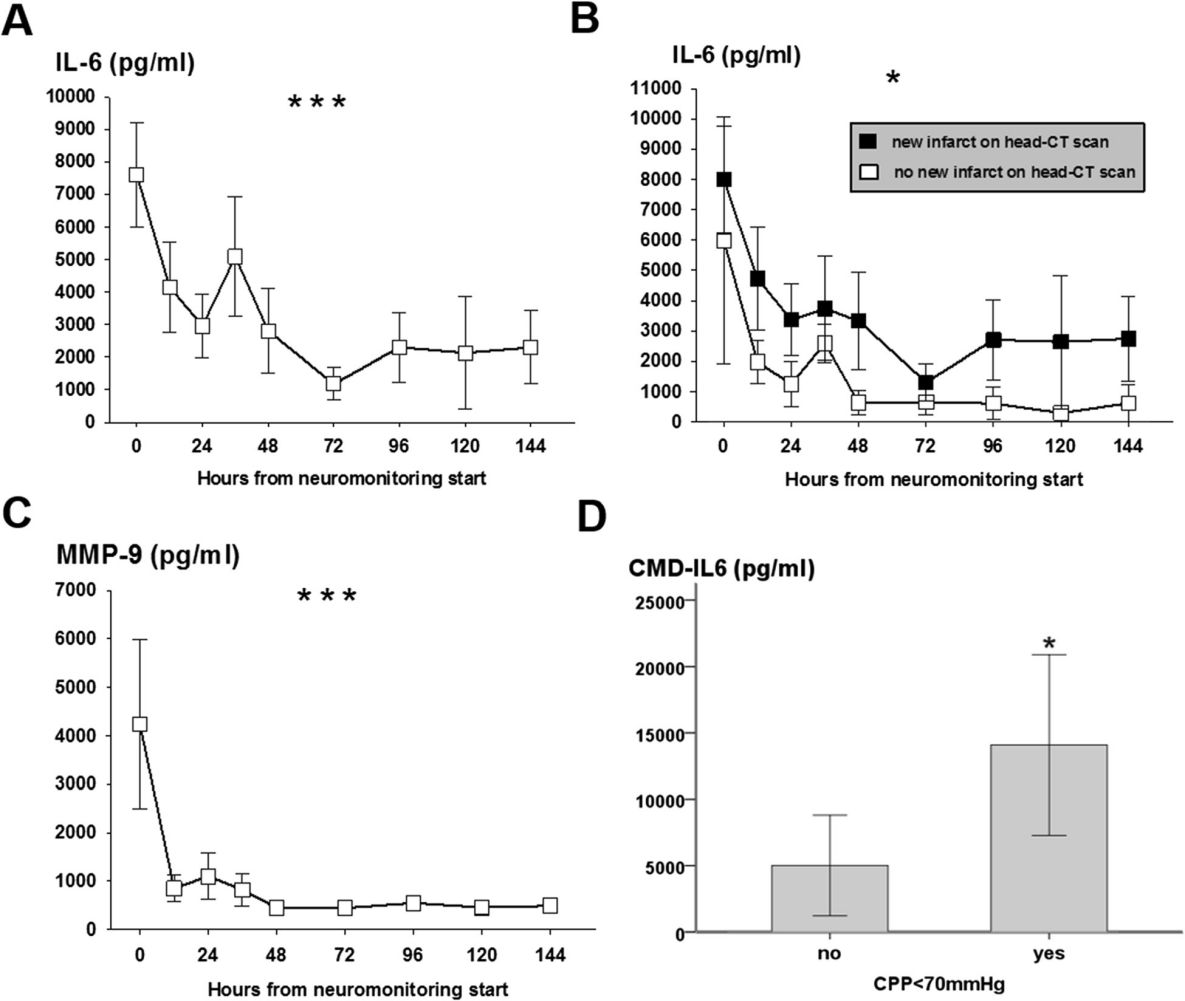
**Mean interleukin-6 (IL-6) and matrix metalloproteinase-9 (MMP-9) levels in the cerebral microdialysate of 26 aneurysmal subarachnoid hemorrhage patients at given time points after neuromonitoring was started.** Significant decrease of **(A)** CMD-IL-6 over neuromonitoring time. Panel **(B)** shows significantly higher levels of CMD-IL-6 in patients developing new infarcts on head computed tomography (CT) scan. Panel **(C)** shows a significant decrease of CMD-MMP-9 over neuromonitoring time. **(D)** Median CMD-IL-6 (interquartile range) levels within the first 12 hours of neuromonitoring in patients with cerebral perfusion pressure (CPP) below (n=9/26, 35%) and above (n=17/26, 65%) 70 mm Hg. CPP is averaged over CMD sampling time. *P <0.05, ***P <0.001. Values are presented as mean±standard error of the mean.

**Figure 4:**
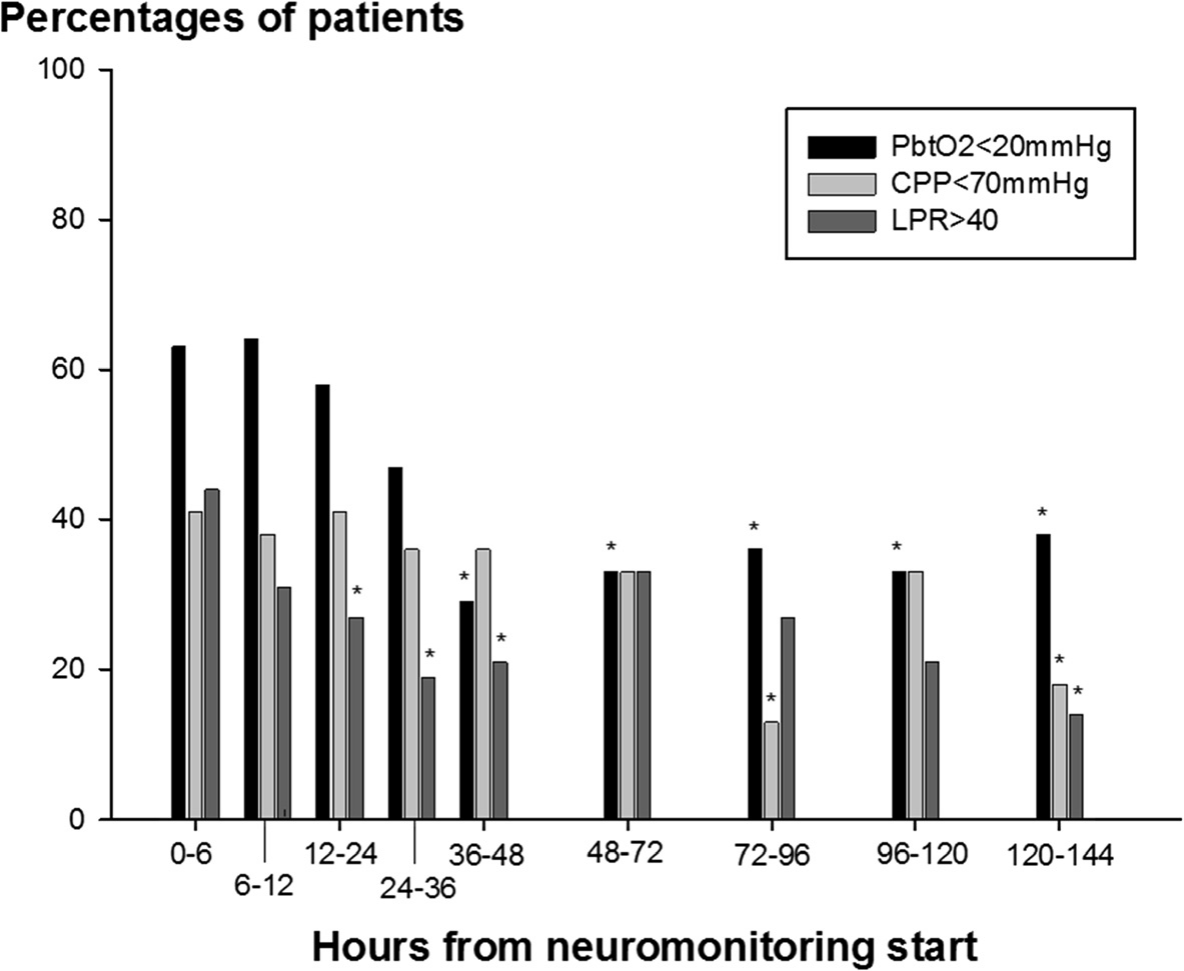
**Percentage of 26 aneurysmal subarachnoid hemorrhage patients with at least one episode (mean hourly value) of brain tissue oxygen tension (P**_**btO2**_ **) below 20 mm Hg, cerebral perfusion pressure (CPP) below 70 mm Hg, and lactate-to-pyruvate ratio (LPR) above 40 in time periods up to 144 hours after neuromonitoring start.** *P <0.05 indicates the significant decrease compared with baseline (baseline = time period 0 to 6 hours from neuromonitoring start).

Continuous variables were assessed for normality. Normally distributed data were reported as mean and standard error of the mean, and non-parametric data were reported as median and interquartile range (IQR). Categorical variables were reported as count and proportions in each group. Hourly recorded concentrations in the cerebral microdialysate were matched to continuously recorded parameters (ICP, CPP, and P_bt_O_2_) averaged over the sampling period (as shown in Figures 1, 2, and 3). Figure 4 displays the percentage of patients with at least one episode (hourly averaged data matched to microdialysis sampling time) in the abnormal range. CMD-derived metabolic parameters and P_bt_O_2_ were categorized as previously defined according to international accepted definitions to associate with CMD-IL-6 and CMD-MMP-9 levels. Time series data were analyzed by using a generalized linear model using a normal distribution and identity-link function and were extended by generalized estimating equations (GEEs) with an autoregressive process of the first order to handle repeated observations within a subject [20]. Data were transformed (log for CMD-IL-6 and CMD-MMP-9) to meet assumptions of normality. In these GEE models, outcome was the dependent variable and important covariates were included (age and admission disease severity). For all tests, significance level was set at a *P* value of less than 0.05. All analyses were performed with IBM-SPSS V20.0 (SPSS Inc., Chicago, IL, USA).

## Results

### General characteristics

Clinical characteristics, hospital complications, and outcome data are summarized in Table 1. Aneurysm was secured within the first 36 hours in all patients by endovascular coiling (n = 8, 31%) or surgical clipping (n = 18, 69%). In half of the patients (n = 13, 50%), CMD catheters were located perilesional; in all other patients, catheters were located in normal appearing brain tissue. Six patients (23%) developed DCI and four patients died during hospitalization (15%).

**Table 1:** **Baseline characteristics, complications, and outcome**

**Clinical characteristics**		**N = 26**
Age, years		55 (47-67)
Gender, female		15 (58%)
Admission H&H grade	2	2 (7.7%)
	3	6 (23.1%)
	4	2 (7.7%)
	5	16 (61.5%)
Loss of consciousness		15 (58%)
Admission APACHE II score		17 (13-19)
Admission radiological characteristics		
mFisher scale	1	3 (11.5%)
	2	3 (11.5%)
	3	9 (34.6%)
	4	11 (42.3%)
SAH sum score		23 (15-27)
IVH Sum score		5 (0-8)
Aneurysm size above 10 mm		7 (27%)
Generalized cerebral edema		11 (42%)
Intracerebral hematoma		12 (46%)
Surgical procedures		
Hydrocephalus requiring EVD/Shunt	20 (77%)	
Clipping		18 (69%)
Hemicraniectomy		9 (35%)
Complications		
Pneumonia		19 (73%)
Delayed cerebral infarction		6 (23%)
Anemia requiring transfusion		16 (62%)
Aneurysm rebleeding		4 (15%)
Hyperosmolar therapy		14 (54%)
Outcome characteristics		
Length of hospital stay, days		40 (30-55)
3-month mRS	0-1	5 (19.2%)
	2-3	5 (19.2%)
	4	5 (19.2%)
	5	6 (23.1%)
	6	5 (19.2%)

Values are expressed as mean (interquartile range) or as number (percentage) of patients. APACHE II, Acute Physiology and Chronic Health Evaluation II; EVD, extraventricular drainage; H&H, Hunt and Hess; ICH, intracerebral hemorrhage; IVH, intraventricular hemorrhage; mFisher, modified Fisher; mRS, modified Rankin Scale; SAH, subarachnoid hemorrhage.

### Cerebrovascular hemodynamics and brain metabolism

Neuromonitoring started at a median of 22 hours after ictus. Mean ICP, CPP, and P_bt_O_2_ were 8 ± 1 mm Hg, 73 ± 2 mm Hg, and 16 ± 3 mm Hg, respectively. ICP remained less than 20 mm Hg and CPP significantly increased from neuromonitoring start to a maximum of 80 ± 2 mm Hg 6 days after ictus (*P* <0.001) (Figure 1A and B) in parallel to mean arterial pressure (*P* <0.001, data not shown). P_bt_O_2_ significantly increased from baseline over the monitoring time (*P* <0.001) (Figure 1C) with at least one episode of brain tissue hypoxia occurring in 63% of patients when neuromonitoring was initiated and decreasing to 29% and 12%, 48 and 96 hours later (Figure 4).

### Brain metabolism and CMD-IL-6 and CMD-MMP-9

Cerebral metabolism revealed a high LPR (42 ± 6) significantly improving over the following days (*P* = 0.002, Figure 2A) secondary to an increase in CMD-pyruvate from initially 113 ± 16 μmol/L to 136 ± 16 μmol/L on day 6 (*P* = 0.04, Figure 2E). CMD-lactate levels remained stable over the neuromonitoring time (Figure 2D). Initially upregulated CMD-glutamate (64 ± 18 mg/dL) decreased over days (*P* = 0.005, Figure 2B). CMD-glucose was normal at the start of neuromonitoring (1.8 ± 0.2 mmol/L) and significantly decreased during hospitalization (*P* = 0.001, Figure 2C) with 53% developing episodes of cerebral glucose of less than 0.7 mmol/L on day 6. Systemic glucose levels were stable over the neuromonitoring time (Figure 2E). CMD-IL-6 levels were above the detection limit in all patients with the highest levels at the start of neuromonitoring (7,600 ± 1,592 pg/mL) and decreased thereafter (*P* <0.001, Figure 3A). Initial highest levels were recorded in patients with aneurysm rebleeding (n = 4, 15%, range 10,500 to 25,300 pg/mL). CMD-MMP-9 was highly elevated (4,238 ± 1,737 pg/mL) at the start of neuromonitoring and significantly decreased within 12 hours to levels below 1,000 pg/mL (*P* <0.001, Figure 3C). There was a correlation between CMD-IL-6 and CMD-lactate (*r* = 0.33, *P* <0.001) and LPR (*r* = 0.18, *P* = 0.01); however, no correlation was found between CMD-IL-6 and CMD-MMP-9 and systemic markers of inflammation (C-reactive protein, absolute leukocyte count, and body temperature) and hemoglobin levels.

### Factors associated with CMD-IL-6 and CMD-MMP-9

Higher CMD-IL-6 levels were recorded initially (first 12 hours of neuromonitoring) in patients with admission GCE (4,440 pg/mL, IQR 805 to 16,194 pg/mL versus 1,542 pg/mL, IQR 155 to 4,059 pg/mL, *P* = 0.02) and patients with a CPP of less than 70 mm Hg (n = 9/26, 35%, 16,281 pg/mL, IQR 6,501 to 21,929 pg/mL versus n = 17/26, 65%, 3,266 pg/mL, IQR 872 to 8,020 pg/mL; *P* = 0.03, Figure 3D) and overall in patients developing DCI independent of disease severity, aneurysm securing method, and perilesional probe location (Wald statistic = 5,4, degrees of freedom (df) = 1, *P* = 0.02, Figure 3B). All other admission variables and hospital complications, including admission H&H grade, aneurysm size of more than 1 cm, the presence of aneurysm rupture-associated intraparenchymal hematoma on admission head CT scan, probe location, hydrocephalus requiring cerebrospinal fluid diversion, and the aneurysm securing method, did not reveal significant differences in CMD-IL-6 levels.

CMD-MMP-9 levels were significantly elevated in the first hours of neuromonitoring in patients who lost consciousness at ictus (*P* = 0.005), H&H grade 5 patients (*P* = 0.002), and patients with initial brain tissue hypoxia (*P* = 0.03) after adjusting for probe location aneurysm repair method and disease severity as appropriate. All other admission and hospital complication were not associated with higher CMD-MMP-9 levels.

### Outcome

CMD-IL-6 levels and LPR were higher in patients with poor 3-month mRS (Wald statistic = 5.7, df = 1, *P =* 0.01 and Wald statistic = 10.1, df = 1, *P =* 0.01). The relationship remained significant after adjusting for age, admission H&H grade, and probe location (Wald statistic = 6.5, df = 1, *P =* 0.01 and Wald statistic = 19.5, df = 1, *P <*0.001). CMD-MMP-9 levels were not associated with poor functional outcome after SAH.

## Discussion

EBI is increasingly recognized to play a key role in pathophysiologic changes contributing to poor functional outcome and mortality after aSAH. Here, we report evidence of brain metabolic derangement, brain tissue hypoxia, neuroinflammation, and BBB disruption in the first 72 hours of neuromonitoring in patients with poor-grade aSAH. Discovering mechanisms of EBI in humans may open the opportunity to target specific treatment endpoints in the early phase after SAH.

Neuroinflammation is increasingly recognized as an innate cerebral response to primary brain injury [21]. In the present study, we did not find an association between higher CMD-IL-6 levels and systemic inflammation, supporting the idea of compartmentalization of the central nervous system.

Pro-inflammatory cytokines may enhance brain edema through disruption of the BBB and induce neuronal apoptosis and therefore directly contribute to early brain damage [22,23]. Cerebral IL-6 has an estimated half-life of several hours and is produced by microglia, astrocytes, and neurons [24]. In previous studies using cerebral microdialysis, the pro-inflammatory cytokine IL-6 was associated with SAH disease severity, the development of DCI, and poor outcome [8-10]. In the present study, we furthermore found an association with admission GCE, metabolic derangement, and a CPP of less than 70 mm Hg. The association between high CMD-LPR and a CPP<70mmHg has been previously reported in SAH patients with admission GCE [7]. Defining the optimal CPP in the early phase after SAH remains a challenge without having predefined brain physiologic endpoints even after aneurysm securing. Brain multimodal monitoring data may be used to target endpoints on the cellular level. In a series of 30 patients with poor-grade SAH, a CPP of less than 70 mm Hg was associated with metabolic distress and brain tissue hypoxia; however, these data cannot be extrapolated to the first 72 hours after SAH [25]. A higher CPP was associated with improved brain metabolism reflected by a lower LPR in a retrospective analysis of aSAH patients with admission GCE [7]. Improving substrate delivery especially in the early phase after SAH may be beneficial in patients with increased need. As shown in patients with traumatic brain injury, CPP augmentation may translate into increased P_bt_O_2_ and a reduction in oxygen extraction fraction [26]. However, a beneficial effect on brain metabolism was not observed. Defining the optimal CPP in the early phase after SAH and identifying patients who may benefit from early augmentation of CPP remain important issues for future research and should include multimodal neuromonitoring data as treatment endpoints.

Another potential treatment target in the early phase after aSAH is to suppress neuroinflammation by the application of systemic anti-inflammatory drugs. Potential benefits in patients with SAH have been postulated [27-29] and are furthermore supported by the improvement of cerebral edema and decreasing neuronal cell apoptosis in experimental SAH models [30]. With the limitation of associated hemodynamic side effects [31] when applied as a rapid infusion, a continuous low-dose infusion may be considered [32].

We found an early upregulation of CMD-MMP-9 in our study population, and higher levels were associated with disease severity, loss of consciousness at ictus, and early brain tissue hypoxia. Loss of consciousness at ictus is highly correlated with poor clinical grade and the development of early or delayed brain edema [18]. MMP-9 contributes to endothelial basal membrane damage, neuroinflammation, and apoptosis and therefore plays a pivotal role in EBI [11-13]. Serum-MMP-9 levels were elevated in patients who developed cerebral vasospasm, although both an initial upregulation and a sustained prolonged increase have been described [15,33]. This again supports the importance of local measurements in the brain as serum markers may reflect a dilution of the innate cerebral response or exaggerated systemic levels originating from multiple organ systems [14,21]. Antagonizing MMP-9 diminished cortical apoptosis, was associated with improved outcome after experimental SAH [34,35], and was recently postulated as potential therapy in ischemic stroke [36].

Bedside analysis of standard metabolic parameters in the cerebral microdialysate revealed a high LPR and an increased release of the excitatory amino-acid glutamate into the extracellular compartment. LPR expresses the redox state of the cell, which is determined by oxygen availability and oxidative metabolism. Glutamate levels were highest at the start of monitoring and gradually returned to near normal baseline values, which has been nicely documented in experimental SAH models [37]. This parallel increased level of LPR is indicative for tissue ischemia and therefore strongly suggestive of global cerebral ischemia in our poor-grade population. However, our monitoring devices were implanted when cerebral recirculation already occurred. Based on pyruvate levels in the normal range in combination with a high LPR, the metabolic profile may also suggest post-ischemic mitochondrial dysfunction, especially in the absence of brain tissue hypoxia 48 hours after ictus. Mitochondrial dysfunction may be diagnosed bedside by using standard metabolic data derived from CMD and was recently investigated in 55 patients with poor-grade SAH [38]. The authors describe a more-than-sevenfold-higher incidence of episodes of mitochondrial dysfunction compared with episodes of cerebral ischemia as cause for disturbed cerebral energy metabolism in patients with SAH [38]. Although no specific treatment to improve mitochondrial dysfunction is currently available, further research is warranted as mitochondrial dysfunction may increase tissue sensitivity to secondary adverse events such as vasospasm and decreased cerebral blood flow.

We observed improvement in brain metabolism and P_bt_O_2_ over the monitoring time most likely secondary to the parallel increase in CPP. Brain extracellular glucose concentrations significantly decreased to a critical level in a substantial amount of patients, whereas systemic glucose levels remained constant and this is suggestive of increased cerebral glucose consumption. Achieving normal cerebral glucose levels should be recommended as neuroglucopenia is associated with metabolic distress and poor outcome after SAH [39].

Quantifying brain metabolism and neuroinflammation may be of importance as both were associated with poor functional outcome. All statistical models were corrected for important covariates, including probe location, as in half of our patients the microdialysis catheter was within 1 cm from the lesion. ‘Perilesional’ probe positioning implies that the microdialysate was collected adjacent to radiological damaged brain tissue, where cell necrosis, blood compounds, autophagy, and apoptosis may alter brain metabolism and ameliorate cytokine release into the extracellular compartment.

Our study was designed as a pilot study and included only a small number of patients and this is a potentially limiting factor. Moreover, early pathophysiologic changes described in the present study may be relevant for patients with poor-grade aSAH and not be generalizable to all clinical grades. We were not able to define specific treatment targets based on the following limitations: (1) a localized metabolic information using cerebral microdialysis technique (2) the small sample size and (3) local treatment strategies which may differ from other institutional protocols and substantially influence longitudinal brain physiologic data. Importantly, patient- and disease-specific data were prospectively documented, and statistical models were corrected for important covariates.

## Conclusions

EBI is believed to substantially contribute to secondary brain injury and to cause significant morbidity and mortality following aSAH. The present study proves that multimodal neuromonitoring techniques can provide insight into pathophysiologic changes in the early phase after aSAH. In our series of 26 patients, catheters were placed within the first 36 hours, revealing metabolic derangement and (to a certain degree) hemodynamic instability, a pro-inflammatory cerebral response, and BBB breakdown. Multimodal neuromonitoring data may assist the neurointensivist in defining treatment targets on the cellular level, eventually opening the door for specific treatment options to minimize early brain injury in patients with aSAH.

## Key messages

- Early brain injury (EBI) is common after subarachnoid hemorrhage (SAH) and is associated with poor outcome.
- Pathophysiologic mechanisms of EBI include blood-brain barrier breakdown, brain tissue hypoxia, neuroinflammation, and excitotoxicity leading to brain edema and metabolic derangement.
- Neuromonitoring techniques may identify underlying pathophysiologic mechanisms occuring in the early phase after aSAH and therefore help to understand mechanisms of EBI.
- Multimodal neuromonitoring data may assist the neurointensivist in defining treatment targets on the cellular level, eventually opening the door for specific treatment options to minimize early brain injury in patients with aneurysmal SAH.

## Abbreviations

aSAH: aneurysmal subarachnoid hemorrhage
BBB: blood-brain barrier
CMD: cerebral microdialysis
CPP: cerebral perfusion pressure
CT: computed tomography
DCI: delayed cerebral ischemia
df: degrees of freedom
EBI: early brain injury
GCE: global cerebral edema
GEE: generalized estimating equation
H&H: Hunt & Hess
ICP: intracranial pressure
IL-6: interleukin-6
IQR: interquartile range
LPR: lactate-to-pyruvate ratio
mBFV: mean blood flow velocity
MMP: matrix metalloproteinase
mRS: modified Rankin Scale
P_bt_O_2_: brain tissue oxygen tension
SAH: subarachnoid hemorrhage
TCD: transcranial Doppler sonography
